# Comparative genomic analysis of *Cohnella hashimotonis* sp. nov. isolated from the International Space Station

**DOI:** 10.3389/fmicb.2023.1166013

**Published:** 2023-06-15

**Authors:** Anna C. Simpson, V. V. Ramprasad Eedara, Nitin K. Singh, Namita Damle, Ceth W. Parker, Fathi Karouia, Christopher E. Mason, Kasthuri Venkateswaran

**Affiliations:** ^1^California Institute of Technology, Jet Propulsion Laboratory, Pasadena, CA, United States; ^2^Department of Plant Science, School of Life Sciences, University of Hyderabad, Hyderabad, India; ^3^Department of Physiology and Biophysics, and the WorldQuant Initiative for Quantitative Prediction, Weill Cornell Medicine, New York, NY, United States; ^4^NASA Ames, Moffet Field, CA, United States

**Keywords:** International Space Station (ISS), novel bacteria, *Cohnella*, space biology, pangenome analysis

## Abstract

A single strain from the family *Paenibacillaceae* was isolated from the wall behind the Waste Hygiene Compartment aboard the International Space Station (ISS) in April 2018, as part of the Microbial Tracking mission series. This strain was identified as a gram-positive, rod-shaped, oxidase-positive, catalase-negative motile bacterium in the genus *Cohnella*, designated as F6_2S_P_1^T^. The 16S sequence of the F6_2S_P_1^T^ strain places it in a clade with *C. rhizosphaerae* and *C. ginsengisoli*, which were originally isolated from plant tissue or rhizosphere environments. The closest 16S and gyrB matches to strain F6_2S_P_1^T^ are to *C. rhizosphaerae* with 98.84 and 93.99% sequence similarity, while a core single-copy gene phylogeny from all publicly available *Cohnella* genomes places it as more closely related to *C. ginsengisoli*. Average nucleotide identity (ANI) and digital DNA–DNA hybridization (dDDH) values to any described *Cohnella* species are <89 and <22%, respectively. The major fatty acids for strain F6_2S_P_1^T^ are anteiso-C_15:0_ (51.7%), iso-C_16:0_ (23.1%), and iso-C_15:0_ (10.5%), and it is able to metabolize a wide range of carbon compounds. Given the results of the ANI and dDDH analyses, this ISS strain is a novel species within the genus *Cohnella* for which we propose the name *Cohnella hashimotonis*, with the type strain F6_2S_P_1^T^ (=NRRL B-65657^T^ and DSMZ 115098^T^). Because no closely related *Cohnella* genomes were available, this study generated the whole-genome sequences (WGSs) of the type strains for *C. rhizosphaerae* and *C. ginsengisoli*. Phylogenetic and pangenomic analysis reveals that F6_2S_P_1^T^, *C. rhizosphaerae*, and *C. ginsengisoli*, along with two uncharacterized *Cohnella* strains, possess a shared set of 332 gene clusters which are not shared with any other WGS of *Cohnella* species, and form a distinct clade branching off from *C. nanjingensis*. Functional traits were predicted for the genomes of strain F6_2S_P_1^T^ and other members of this clade.

## Introduction

The International Space Station (ISS) is an orbital space habitat which has been continuously inhabited by astronauts for 22 years (Robinson et al., 2006) and is therefore the only current model for how the microbiome of a space habitat evolves over time. Because the ISS is hermetically sealed against the vacuum of space, sources of its microbiome must necessarily be the human commensals from the astronauts, new equipment and supplies, or experiments conducted on board (Checinska Sielaff et al., 2019; Urbaniak et al., 2022). With a number of both governmental and commercial closed-habitat space facilities planned for both earth and lunar orbit in the near future, studying the microbiome of this unique habitat is vital to understanding both how astronaut health is affected by the presence of bacteria and fungi exposed to the space environment, and how those microbes can be utilized for biotechnological applications. Part of this effort should include the whole-genome sequencing (WGS) and taxonomic classification of microbes isolated from the ISS, to track their sources, spread, interactions, and potential applications. Until the mid-2010s, monitoring of bacteria and fungi aboard the ISS was mainly done via traditional culturing and identification using Sanger sequencing methods, to ensure crew health and food and water safety (La Duc et al., 2004; Checinska et al., 2015). More recently, a series of NASA Space Biology-funded missions known as Microbial Tracking 1 and 2 investigated the metagenomes of ISS surfaces and performed WGS on all isolated bacteria and fungi (Checinska Sielaff et al., 2016; Venkateswaran et al., 2017; Urbaniak et al., 2018; Bijlani et al., 2020; Daudu et al., 2020; Solomon et al., 2020; Blachowicz et al., 2021; Simpson et al., 2021a,b), in order to fully characterize the ISS microbiome and how it changes over time (Singh et al., 2018; Checinska Sielaff et al., 2019; Urbaniak et al., 2022). In the ongoing Microbial Tracking investigation of the ISS, spore-forming bacteria have been isolated multiple times from the genera *Bacillus* and *Paenibacillus*; however, this is the first instance that a bacterium from the genus *Cohnella* has been isolated from an ISS surface. As part of the Microbial Tracking 2 mission series, during April of 2018, astronauts sampled various surfaces aboard the ISS (Urbaniak et al., 2022) with pre-packaged, pre-sterilized wipes, including the panel behind the Waste Hygiene Compartment (WHC). Upon return to Earth, the strain F6_2S_P_1^T^ was isolated from the WHC sample wipe, on blood agar incubated at 37°C. Preliminary 16S rRNA sequence analysis placed strain F6_2S_P_1^T^ in the genus *Cohnella*.

The genus *Cohnella* (family *Paenibacillaceae*, order *Bacillales*, phylum Firmicutes/Bacillota) was first proposed by Kampfer et al. (2006) as distinct from the genus *Paenibacillus*, with the description of *Cohnella thermotolerans* (type species, isolated during hygiene checks at a starch production company in Sweden) and the reclassification of *Paenibacillus hongkongensis* as *Cohnella hongkongensis* (Teng et al., 2003). *Cohnella* species are differentiated from *Paenibacillus* due to high iso-C_16:0_ content in their fatty acid profiles (Kampfer et al., 2006). Members of *Cohnella* are rod-shaped, usually endospore-forming, and gram-positive.

*Cohnella* species have been first isolated from a wide variety of environmental samples, most usually in soil (Kampfer et al., 2014), but also in plant tissue (Garcia-Fraile et al., 2008; Flores-Felix et al., 2014) and compost heaps (He et al., 2013). Species from this genus are used in industrial applications including fermentation, biofuel production, and wastewater treatment bioreactors (Lin et al., 2020; Hero et al., 2021; Chen et al., 2022; Mohammadi et al., 2022). As with the genus *Paenibacillus*, many members of the genus *Cohnella* are strongly associated with the plant rhizosphere environment, particularly that of legume species (Niang et al., 2018). Multiple novel *Cohnella* species have been discovered in the root nodules of leguminous plants, although only two species are known to fix nitrogen (Garcia-Fraile et al., 2008; Wang et al., 2012, 2015; Flores-Felix et al., 2014; Takada et al., 2019). *Cohnella* species are also thought to play an important role in recycling plant biomass within soil, with multiple members of the genus possessing genes for degradation of hemicellulose, cellulose, and xylan (Khianngam et al., 2010; Hameed et al., 2013; Hero et al., 2021; Johnson et al., 2021). Overall, while *Cohnella* species have been occasionally detected in the blood and oral cavities of immune-compromised or otherwise unhealthy individuals (Teng et al., 2003; Abou Abdallah et al., 2019; Kim et al., 2021), they do not appear to be common human commensals.

In this study, we completed the characterization study of strain F6_2S_P_1^T^ and describe it here with three main objectives. The first objective was to establish *Cohnella* sp. F6_2S_P_1^T^ as a novel species, for which chemotaxonomic, phenotypic, phylogenetic (using taxonomic marker genes), and comparative genomic analyses were carried out. In addition, initial taxonomic analysis for strain F6_2S_P_1^T^ showed that it was most closely related to four *Cohnella* species without publicly available genomes, two of which are officially recognized species (Kim et al., 2010; Kampfer et al., 2014). Thus, the strains *Cohnella ginsengisoli* DSM 18997^T^ and *Cohnella rhizosphaerae* DSM 28161^T^ were acquired from the Deutsche Sammlung von Mikroorganismen und Zellkulturen (DSMZ) and whole-genome-sequenced, and assembled draft genomes were generated for these species.

The second objective was to discover the phylogenetic placement of strain F6_2S_P_1^T^ within the genus *Cohnella*, for which we performed multiple phylogenetic analyses as well as a pangenome, and a genome-based tree containing all shared single-copy core genes from the genus *Cohnella*. The third objective was to explore the functional traits predicted by the genome of strain F6_2S_P_1^T^. Because strain F6_2S_P_1^T^ is part of a branch of the genus *Cohnella* which is only distantly related to the rest of the genus, the functional traits predicted for the entire closely related clade of which strain F6_2S_P_1^T^ is a member were characterized.

## Materials and methods

### Sample collection and isolation

Methods for preparing sampling kits and processing samples were similar to those used in the Microbial Tracking 1 mission (Singh et al., 2018; Checinska Sielaff et al., 2019). For each of five flight missions for Microbial Tracking 2, Sterile TexTra™ 10 TX3224 Dry Cleanroom Wipers were moistened with 15 ml of sterile phosphate-buffered saline (PBS), folded into quarters and placed in sterile Ziploc baggies, which were later used to assemble sampling kits at NASA Ames. Sampling kits were transported to the ISS, where astronauts collected surface particulates from a square meter area of eight surfaces: (1) the cupola, (2) the WHC, (3) the advanced resistive exercise device, (4) dining table, (5) Zero-Gravity Stowage Rack overhead 4, (6) permanent multipurpose module Port 1, (7) laboratory overhead 3, and (8) crew quarters bump-out exterior wall (see Singh et al., 2018 for detailed surface descriptions). For each surface, the astronaut tasked with sampling donned a new pair of sterile gloves, removed and unfolded a sterile wipe from its bag, and wiped a square meter area of the surface three times, folding the wipe in half for the second pass and into quarters for the third pass; each time, the surface was sampled in a different direction (horizontally, vertically, and diagonally) using the new previously unexposed surface of the wipe. Additionally, a wipe was removed from its bag and briefly exposed to the air, as an environmental control. A total of nine (eight locations and one control) samples were produced for each flight and stored at 4°C aboard the ISS and during transport to earth.

Upon delivery to Jet Propulsion Laboratory, ISS wipes and unopened control wipes were processed for the isolation of environmental microbes within a 12-h period. Each wipe was placed in a sterile 500-ml bottle with 200-ml sterile PBS and shaken vigorously for 2 min. PBS was then concentrated using an Innovaprep CP-150 (Innovaprep LLC, Drexel, Missouri, United States) with a 0.2 μm polysulfone hollow fiber concentrating pipette tip (Innovaprep LLC, SKU CC08022-10). Using additional sterile PBS, dilutions of 10^−1^ through 10^−6^ were prepared and plated onto Reasoner's 2 agar (R2A, BD Diagnostics Cat # 218263), potato dextrose agar (BD Diagnostics Cat #213400) with 0.05 g/L chloramphenicol, and blood agar (Hardy Diagnostics, #A10) media. Five colonies from each sample on each media type were selected and stored in one-tenth strength tryptic soy agar (TSA) stabs. Strain F6_2S_P_1^T^ was isolated from blood agar inoculated with the PBS concentrate from Location 2 (wall behind WHC), collected during Flight 6 (third flight in the Microbial Tracking 2 mission series).

### DNA extraction and whole-genome sequencing

The ZymoBIOMICS DNA MagBead kit was used to extract genomic DNA from strain F6_2S_P_1^T^ according to the manufacturer's instructions. An Illumina Nextera DNA Flex library preparation kit was used to prepare the library for WGS as in Simpson et al. (2021b). The library was sequenced as part of a run on a NovaSeq 6000 S4 flow cell paired-end 2 × 150-bp platform. FastQC v0.11.7 (Bioinformatics, 2011) was used to quality filter and trim reads, and fastp v0.20 (Chen et al., 2018) was used to remove adapter sequences. A draft genome was assembled to the scaffold level using SPAdes v3.11.1 (Bankevich et al., 2012). Assembly quality, including number of contigs, genome size, and N50 value, was assessed using QUAST v5.0.2 (Gurevich et al., 2013). Default settings were used for all steps except for fastp, which included 512 adapters screening.

A secondary round of sequencing for strain F6_2S_P_1^T^, as well as *C. ginsengisoli* DSM 18997^T^ and *C. rhizosphaerae* DSM 28161^T^, was conducted using Oxford Nanopore sequencing. *C. ginsengisoli* DSM 18997^T^ and *C. rhizosphaerae* DSM 28161^T^ were ordered from the DSMZ and cryostocks prepared for long term storage. To produce a more resolved genome for *Cohnella* sp. F6_2S_P_1^T^ and to provide WGS, all strains were re-streaked from cryostock on TSA and genomic DNA was extracted using a Zymo QuikSpin DNA extraction kit. Nanopore sequencing libraries were prepared using an SQK-LSK110 Ligation Sequencing kit (Oxford Nanopore) and sequenced on an R9.4.1 flow cell, with base calling via MinKNOW/Guppy. Porechop (Wick et al., 2017a) and Nanofilt (De Coster et al., 2018) were used to remove adapter sequences and filter reads using the settings “-q 10 -l 200.” A hybrid genome assembly for *Cohnella* sp. F6_2S_P_1^T^ was generated via Unicycler (Wick et al., 2017b) using Nanopore reads to polish the Illumina assembly. Flye (Kolmogorov et al., 2019) followed by polishing with Medaka (Oxford Nanopore Technologies) was used to assemble draft genomes for type strains of *C. ginsengisoli* and *C. rhizosphaerae* from Nanopore reads, and assembly quality was assessed using QUAST v5.0.2 (Gurevich et al., 2013).

### Phylogenetic analysis

Phylogenetic analysis using 16S rRNA gene sequences was carried out by extracting the 16S rDNA sequence from the WGS of strain F6_2S_P_1^T^ and aligning it to the NCBI nucleotide database via blast search. The top 30 matching 16S rRNA sequences were retrieved. In addition, all publicly available genomes from the genus *Cohnella* were downloaded, and their 16S rDNA sequences were extracted using a blast wrapper script. 16S rRNA genes from the species *Paenibacillus barengoltzii, Paenibacillus terrae, Thermoflavimicrobial dichotomicus*, and *Thermoactinomyces vulgaris* were used to root the tree. Similarly, gyrB sequences from strain F6_2S_P_1^T^ and from all publicly available *Cohnella* genomes, and the outgroups *T. dichotomicus and T. vulgaris* were extracted with the same wrapper script. For both gyrB and 16S trees, alignment was performed in R using the DECIPHER package (Wright, 2016). Trimming and tree-building using maximum likelihood and the substitution model GTR + G + I were performed using the phangorn package, including model selection using AIC values and 1,000 bootstrap replicates (Schliep, 2011). Phylogenetic trees based on 16S rRNA and gyrB genes were visualized using ggtree (Yu et al., 2017).

A genome-based phylogenetic tree was also generated using genes detected from the pangenomic analysis, which contained all publicly available *Cohnella* genomes, plus those of strain F6_2S_P_1^T^, *C. ginsengisoli*, and *C. rhizosphaerae* generated during this study (see below section for methods for pangenomic tree generation). Aligned, single-copy core genes which were shared by all species and had a geometric homogeneity of 1, but a functional homogeneity below 0.9, were extracted from the *Cohnella* genus pangenome into a concatenated FASTA file. A maximum likelihood genome-based tree was generated using raxml v8.2.12 (Stamatakis, 2014; Kozlov et al., 2019) with a GTR-GAMMA substitution model and the option “autoMRE” to automatically choose the optimal number of bootstrap replicates. The tree was visualized using the online platform Interactive Tree of Life (iTOL) (Letunic and Bork, 2007).

Additionally, average nucleotide identity (ANI) and digital DNA–DNA hybridization (dDDH) were calculated for strain F6_2S_P_1^T^ against all sequenced and described *Cohnella* species using fastANI v1.33 (Jain et al., 2018) and DSMZ's online Genome-to-Genome Distance Calculator v3.0 (Meier-Kolthoff et al., 2022).

### Light microscopy and SEM

A liquid culture of strain F6_2S_P_1^T^ was incubated at 80°C for 10 min, then plated on R2A and grown at 30°C for 5 days to induce endospore formation. Endospore staining with malachite green and safranin was performed using the Schaeffer–Fulton method (Schaeffer and Fulton, 1933). Light microscopy and phase contrast images were taken on an Olympus BX53 microscope with an Olympus DP25 camera using Olympus cellSens software.

To prepare a colony of strain F6_2S_P_1^T^ for SEM, the strain was streaked out on TSA and allowed to grow at 30°C for 48 h. A pure colony was then suspended in chilled 2.5% glutaraldehyde (Ted Pella Inc., Redding, CA, United States) in 0.1M sodium cacodylate (NaCaco) (Sigma Aldrich) solution for 1 h. The sample was then filtered through a 0.2 μm Isopore filter membrane (MilliporeSigma, Burlington, MA, United States) and moved into a 1.5 ml centrifuge tube. The sample was then incubated in 0.1 M NaCaco solution at 4°C for 10 min and then replaced with fresh 0.1 M NaCaco solution; this replacement was repeated three times. The sample was then dehydrated in a series of increasing IPA solutions mixed with water for 10 min each. The series was 50, 70, 80, 90, 95, and 100%, with the final 100% IPA rinsing occurring three times. The specimen was stored at 4°C in 100% IPA until it was critical point dried in a Tousimis Automegasamdri 915B critical point dryer (Rockville, MD, United States). Samples were adhered with carbon tape to SEM stubs (Ted Pella Inc.) and then underwent ~12 nm thick carbon coating with a Leica EM ACE600 Carbon Evaporator (Deerfield, IL, United States). SEM micrographs were captured on a FEI Quanta 200F microscope (Thermo Fisher, Waltham, MA, United States) located at the California Institute of Technologies Kavli Nanoscience Institute.

### Pangenomic analysis and other genome characterization

Pangenomic analysis was carried out using anvi'o v7.1 (Eren et al., 2015) similar to methods described in Singh et al. (2021). Open reading frames were annotated with COG, KEGG, and KOfam database pathways/functions, and ANI between all genomes was additionally calculated. Two pangenomes were generated: one including all publicly available *Cohnella* genomes including unnamed species and one containing only named species. The function *anvi-compute-functional-enrichment-in-pan* was used to calculate functional enrichment in the *C. ginsengisoli* clade compared to the rest of the genus *Cohnella*.

The genome of the strain F6_2S_P_1^T^ was also uploaded and annotated using the online Rapid Annotation using Subsystems Technology (RAST platform) (Aziz et al., 2008). The presence of biosynthetic gene clusters (BGCs) which might produce secondary metabolites was predicted in the strain F6_2S_P_1^T^ genome using the antiSMASH pipeline (Blin et al., 2021). The presence of antimicrobial resistance or virulence genes was predicted by the ABRicate pipeline (Seemann, 2018).

To evaluate the diversity of carbohydrate-degrading and other carbohydrate-cycling enzymes (Carbohydrate-Active Enzymes, i.e., CAZymes) (Drula et al., 2022), the dbcan3 stand-alone tool (Zhang et al., 2018) was run against all publicly available genomes in the genera *Cohnella* and *Paenibacillus* as well as the genomes of strain F6_2S_P_1^T^, *C. ginsengisoli*, and *C. rhizosphaerae*. The results were parsed to generate a count matrix for the number of each CAZyme family/subfamily predicted in the genome of each species. All statistics and plots from analysis of this data were generated in R [v 4.2.2, “Innocent and Trusting,” R Core Team (2022)] utilizing the Tidyverse package (Wickham et al., 2019). An NMDS ordination (stress = 12.9, *k* = 3) was generated via vegan (Oksanen et al., 2019) using Bray–Curtis distance after standardization of each CAZyme category to between 0 and 1, and individual Kruskal–Wallis tests were performed for each CAZyme testing mean abundance between members of the *C. ginsengisoli* clade and other species of *Cohnella* and *Paenibacillus* with Bonferroni correction for multiple testing. Additionally, a heatmap of abundance for each category was generated using the pheatmap package (Kolde and Kolde, 2018) after standardization of the data using robust centered log ratios.

### Phenotypic characterization

Growth temperature was determined by inoculating both solid (R2A plates) and liquid (trypticase soy broth (TSB), BD Diagnostics Cat # 257107) media in 15 ml loose-capped centrifuge tubes with strain F6_2S_P_1^T^ and growing at 4, 15, 25, 30, 37, and 45°C. Plates and tubes were checked for growth daily for 7 days and incubation was halted if growth was observed. Samples grown at 4 and 15°C were incubated for additional time before final growth assessment: 2 weeks total for growth at 15°C and 4 weeks total at 4°C. Salt tolerance was determined by inoculating strain F6_2S_P_1^T^ onto R2A with 0, 1, 2, 3, 4, and 5% added NaCl, as well as agar containing only peptone plus 0 or 1% NaCl, and examining growth after 7 days of incubation at 30°C. Oxidase activity was determined by testing solid culture with OxiDrops™ liquid oxidase reagent (Hardy Diagnostics). Catalase activity was determined by mixing colonies of strain F6_2S_P_1^T^ with hydrogen peroxide on a sterile glass slide and monitoring for the presence of effervescence. pH tolerance (4–10) was tested by adjusting the pH of TSB broth with biological buffers as in Xu et al. (2005).

### Mapping of metagenomic reads to genome

Metagenomic reads from ISS surfaces from Microbial Tracking 1 (Checinska Sielaff et al., 2019) and Microbial Tracking 2 (Urbaniak et al., 2022) were mapped to the genome of *Cohnella* sp. F6_2S_P_1^T^ using Bowtie2 (Langmead and Salzberg, 2012), using the flags “—no-mixed” (no unpaired alignments) and “—no-discordant” (only matches where both forward and reverse reads map to the same area are considered). The number of matches where both a forward and reverse read mapped to the *Cohnella* sp. F6_2S_P_1^T^ genome were reported as a percentage of total reads.

### Chemotaxonomic analysis

Cells grown in TSB medium were harvested when growth of the cultures reached approximately 70% of the maximal optical density (exponential growth phase) and used for analysis of cellular fatty acids, polar lipids, and quinones, which was done as described previously (Ramaprasad et al., 2015). In brief, for cellular fatty acids analysis, 40 mg of bacterial cells were subjected to a series of four different reagents followed by saponification and methylation of fatty acids, thus enabling their cleavage from lipids. The fatty acid methyl esters thus obtained were analyzed by gas chromatography. The peaks obtained were then labeled and the equivalent chain length values were computed as published elsewhere (MIDI, 2012).

The polar lipid profile was analyzed by extracting polar lipids with methanol/chloroform/saline (2:1:0.8, by vol.) from 1 g freeze-dried bacterial cells. Separation of lipids was done by two-dimensional chromatography on a silica gel TLC plate (Kieselgel 60 F254; Merck) using chloroform–methanol–water (75:32:4 by vol.) in the first dimension and chloroform–methanol–acetic acid–water (86:16:15:4 by vol.) in the second dimension. Total polar lipid profiles were detected by spraying with 6% ethanolic molybdophosphoric acid. The respiratory isoprenoid quinone was extracted with chloroform/methanol mixture (2:1, v/v), evaporated under a vacuum, re-extracted with acetone, and analyzed using high-performance lipid chromatography according to previously reported methods (Ramaprasad et al., 2018). Polyamines were analyzed as described by Busse and Auling (1988) and Busse et al. (1997). The peptidoglycan of cells of strain F6_2S_P_1^T^ was isolated following disruption of the cells by shaking with glass beads and subsequent trypsin digestion, according to the method of Schleifer (1985). Cell wall was hydrolyzed for amino acid analyses as described by Schleifer and Kandler (1972). Amino acids in cell-wall hydrolysates were analyzed by high performance liquid chromatography (HPLC) (Mckerrow et al., 2000; Ramaprasad et al., 2015). Carbon substrate utilization profile analysis was carried out as per the BioLog protocol provided by the manufacturer for Firmicutes using the GEN III MicroPlate test assay with a Biolog system (Hayward, CA, USA).

## Results and discussion

*Cohnella* sp. F6_2S_P_1^T^ isolated from the ISS was subjected to polyphasic taxonomic characterization to determine its phylogenetic affiliation. In addition, WGSs of *C. ginsengisoli* and *C. rhizosphaerae* were generated and compared with the genome of *Cohnella* sp. F6_2S_P_1^T^ to identify the variable, conserved, and distinctive genomic traits.

### Genome characteristics and relatedness indices

Assembly statistics for *Cohnella* sp. F6_2S_P_1, *C. ginsengisoli*, and *C. rhizosphaerae* are summarized in Table 1. Genome sizes are 9.06 Mb for strain F6_2S_P_1, 7.22 Mb for *C. ginsengisoli*, and 8.5 Mb for *C. rhizosphaerae*. GC content ranged from 58.37% for *Cohnella* sp. F6_2S_P_1^T^ to 59.49% for *C. rhizosphaerae*. In general, among 39 sequenced *Cohnella* genomes, genome size varied from 4.0 to 9.4 Mb. This genome variation should be investigated for any functional genes acquired or shed over time.

**Table 1:** Assembly statistics for hybrid Illumina and Nanopore assembly of *Cohnella* sp. F6_2S_P_1^T^.

**Species/strain**	**NCBI accession no**.	**Isolation location**	**No. of scaffolds/ contigs**	**Genome size (bp)**	**N50 (bp)**	**Average coverage**	**G + C content (%)**	**Filtered reads used for assembly**	**Coding sequences**
*Cohnella hashimotonis* F6_2S_P_1^T^	JAGRPV000000000	Waste hygiene compartment, ISS	8	9,059,145	8,667,868	368	58.37	34,984,402 (Illumina), 312,000 (Nanopore)	7,569
*Cohnella ginsengisoli* DSM 18997^T^	JAPDHZ000000000	Ginseng soil (Kim et al., 2010)	8	7,220,644	2,121,708	57	59.06	184,000 (Nanopore)	6,543
*Cohnella rhizosphaerae* DSM 28161^T^	JAPDIA000000000	Maize rhizosphere (Kampfer et al., 2014)	9	8,497,463	2,277,727	55	59.48	160,000 (Nanopore)	7,541

ANI values between *Cohnella* sp. F6_2S_P_1^T^ and *C. ginsengisoli*, and *C. rhizosphaerae* were 88.4 and 88.0%, respectively (Table 2). ANI between *Cohnella* sp. F6_2S_P_1^T^ and all other named *Cohnella* species for which a genome was publicly available was <79.4% (Table 2). We also tested ANI between *Cohnella* sp. F6_2S_P_1^T^ and available genomes for unnamed *Cohnella* species and found that *Cohnella* sp. OV330 and *Cohnella* sp. GbtcB17 were also closely related, with ANIs of 88.9 and 88.0%, respectively. This was also the trend for *C. ginsengisoli* and *C. rhizosphaerae*, which showed ANI to each other and to *Cohnella* sp. OV330 and *Cohnella* sp. GbtcB17 ranging from 88 to 92%, and which was otherwise most closely related to *C. nanjingensis* with an ANI value of ~79%. The dDDH results showed a similar pattern, as values were 35 and 34.5% between *Cohnella* sp. F6_2S_P_1^T^ and *C. ginsengisoli*, and *C. rhizosphaerae*, respectively, and were otherwise below 22% (Table 2).

**Table 2:** Average nucleotide identity and digital DNA hybridization values between strain F6_2S_P_1^T^ and other named members of the genus *Cohnella*.

	***Cohnella hashimotonis*** **F6_2S_P_1**^**T**^	
**Species (NCBI accession no.)**	**ANI value (%)**	**dDDH (%) (formula 2)**
*Cohnella ginsengisoli* DSM 18997^T^	88.4	35
*Cohnella rhizosphaerae* DSM 28161^T^	88.0	34.2
*Cohnella nanjingensis* DSM 28246^T^ (GCA_014212125.1)	79.3	21.5
*Cohnella xylanilytica* DSM 25239^T^ (GCA_014212175.1)	78.6	20.3
*Cohnella thailandensis* DSM 25241^T^ (GCA_014212135.1)	78.3	20.2
“*Cohnella zeiphila*” CBP-2801 (GCA_014212055.1)	78.2	19.9
*Cohnella lubricantis* DSM 103658^T^ (GCA_014212015.1)	78.2	20.3
“*Cohnella candidum*” 18JY8-7 (GCA_003713065.1)	78.0	20.1
*Cohnella thermotolerans* DSM 17683^T^ (GCA_000429825.1)	78.0	19.9
*Cohnella algarum* Pch-40^T^ (GCA_016937515.1)	77.7	20.5
*Cohnella laeviribosi* DSM 21336^T^ (GCA_000378425.1)	77.5	19.5
*Cohnella panacarvi* Gsoil 349^T^ (GCA_000515335.1)	77.5	19.7
“*Cohnella abietis”* HS21 (GCA_004295585.1)	<77	22.6
*Cohnella boryungensis* DSM 26489^T^ (GCA_016908615.1)	<77	19
*Cohnella endophytica* M2MS4P-1^T^ (GCA_003628305.1)	<77	19.7
*Cohnella faecalis* K2E09-144^T^ (GCA_003570905.1)	<77	20.4
*Cohnella kolymensis* VKM B-2846^T^ (GCA_000829465.1)	<77	18.9
*Cohnella luojiensis* CCTCC AB 208254^T^ (GCA_004564235.1)	<77	18.5
*Cohnella lupini* CECT 8236^T^ (GCA_003386205.1)	<77	19.4
*Cohnella phaseoli* CECT 7287^T^ (GCA_003386235.1)	<77	20.6
“*Cohnella pontilimi”* CAU 1483 (GCA_005049045.1)	<77	19.4

### Phylogeny of strain F6_2S_P_1^T^

**Figure 1:**
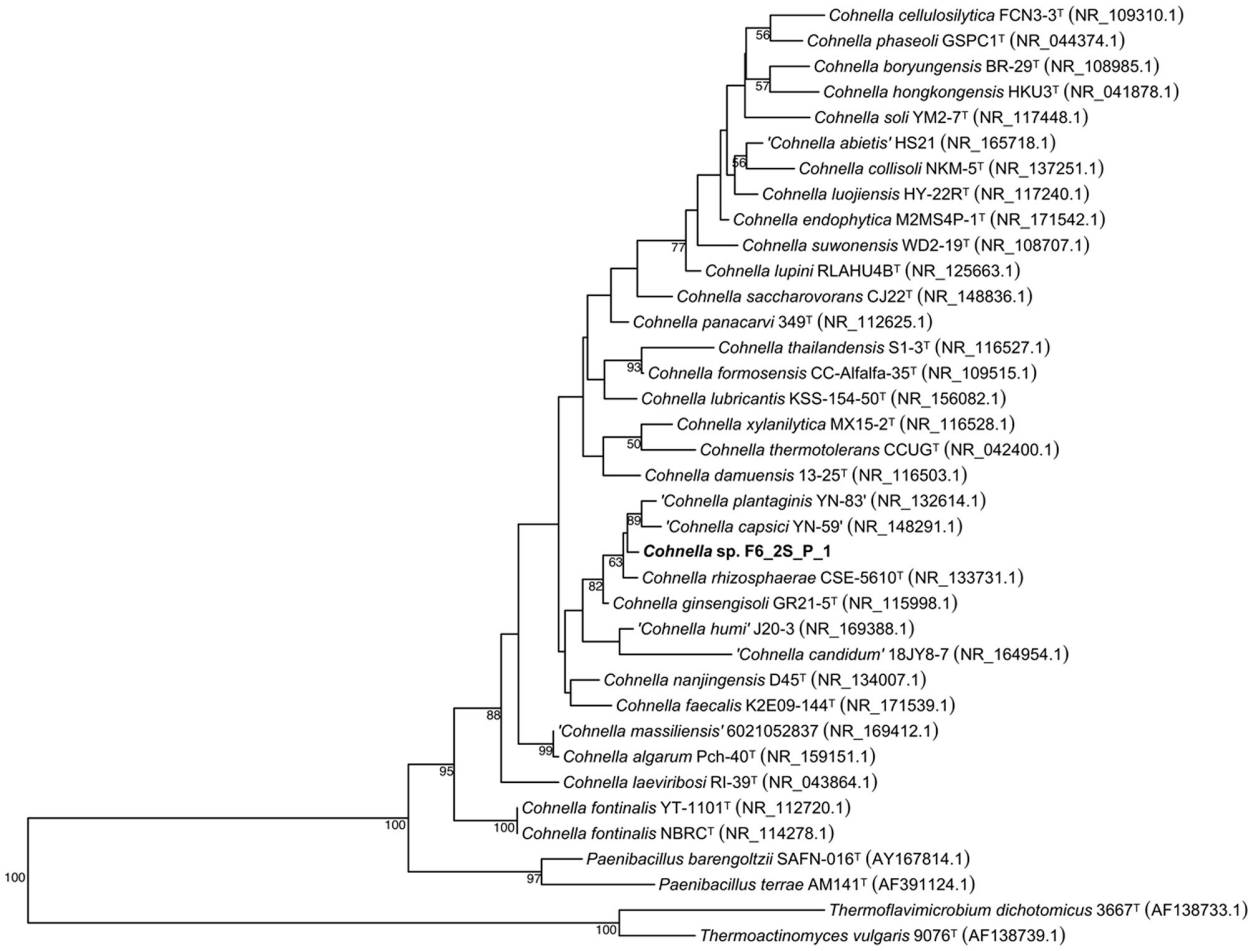
Maximum likelihood phylogenetic tree based on 16S rRNA gene sequences, showing the inclusion of Cohnella sp. F6_2S_P_1^T^ in a branch of the genus including species isolated from plant tissue or rhizosphere soil.

**Figure 2:**
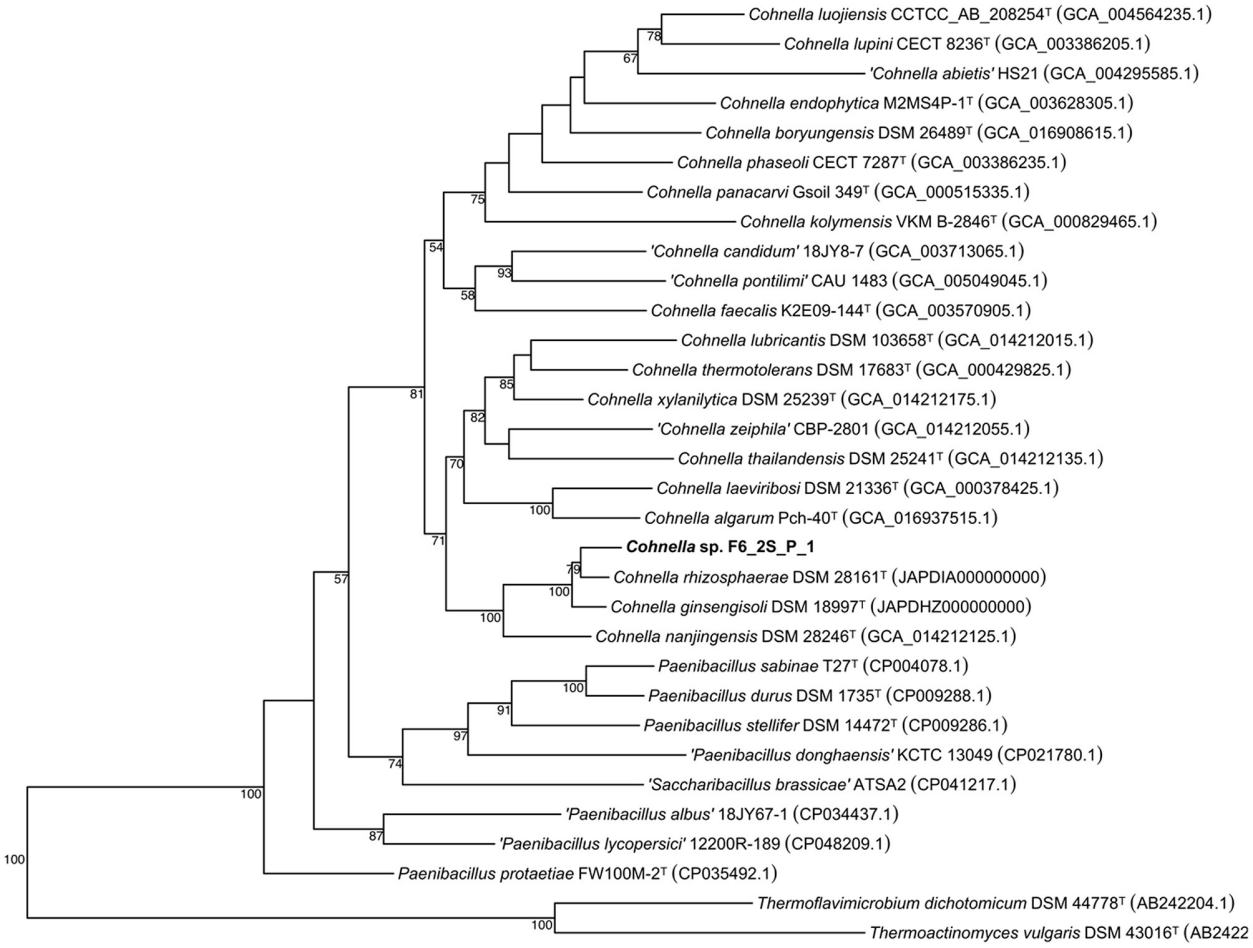
Maximum likelihood phylogenetic tree based on gyrB sequences of the genus *Cohnella*.

A 16S rDNA-based phylogenetic tree of all *Cohnella* species, plus the species *P. barengoltzii, P. terrae, T. dichotomicus, and T. vulgaris* as outgroups, shows that strain F6_2S_P_1^T^ is placed within a clade containing the officially named species *C. rhizosphaerae* and *C. ginsengisoli*, as well as yet-to-be described species “*C. plantaginis”* and “*C. capsici”* (Figure 1). The 16S rDNA sequence similarities of strain F6_2S_P_1^T^ with the members of this clade ranged from 98.84 to 97.37%. “*C. plantaginis”* and “*C. capsici”* form their own well-supported branch within this clade, while *C. rhizosphaerae* is basal to strain F6_2S_P_1^T^ and *C. ginsengisoli* is the most basal species of the clade (Figure 1). However, branch support for the divergence of strain F6_2S_P_1^T^ from *C. rhizosphaerae* was low (50) and not shown in Figure 1. A gyrB-based phylogeny was also generated using all publicly available *Cohnella* genomes plus the outgroups *T. dichotomicus and T. vulgaris* (Figure 2). The *gyrB* sequence similarities of strain F6_2S_P_1^T^ with the members of this clade ranged from 93.99 to 93.55%. The *gyrB* tree supports the position of strain F6_2S_P_1^T^ as non-basal to *C. rhizosphaerae* and *C. ginsengisoli*, with *C. ginsengisoli* as the most basal species in this branch of the genus *Cohnella*. Since WGSs of “*C. plantaginis”* and “*C. capsici”* are not available, they were not included in the gyrB phylogeny.

**Figure 3:**
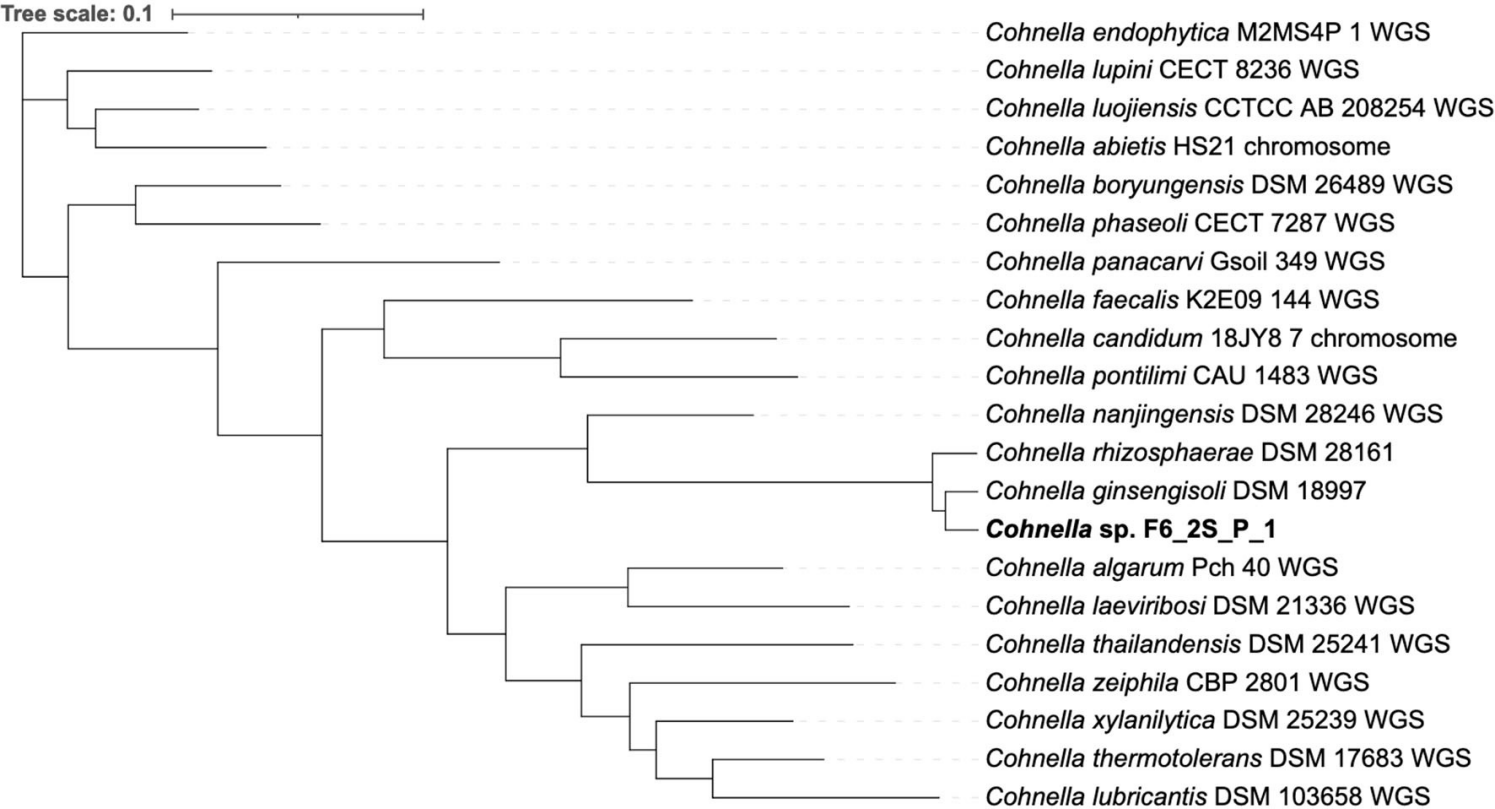
Phylogenetic tree based on 294 single-copy core genes shared by all available *Cohnella* species genomes.

The genome-based tree (Figure 3) for all sequenced and fully described *Cohnella* species was based on a concatenated alignment of 14 gene clusters containing a total of 294 single-copy core genes common to all species in the genus. This tree supports the position of *C. rhizosphaerae* as basal to *C. ginsengisoli*, in contrast to the 16S and gyrB trees. Nevertheless, the position of strain F6_2S_P_1^T^ remains the same. A second genome-based tree was generated using a second pangenome analysis including genomes of all *Cohnella* species along with yet-to-be described species (Supplementary Figure S1), which showed that *Cohnella* sp. OV330 and *Cohnella* sp. GbtcB17 are also part of this clade, with *Cohnella* sp. GbtcB17 as the most basal species and *Cohnella* sp. OV330 as basal to *C. ginsengisoli* and strain F6_2S_P_1^T^.

### Phenotypic and chemotaxonomic characterization

**Figure 4:**
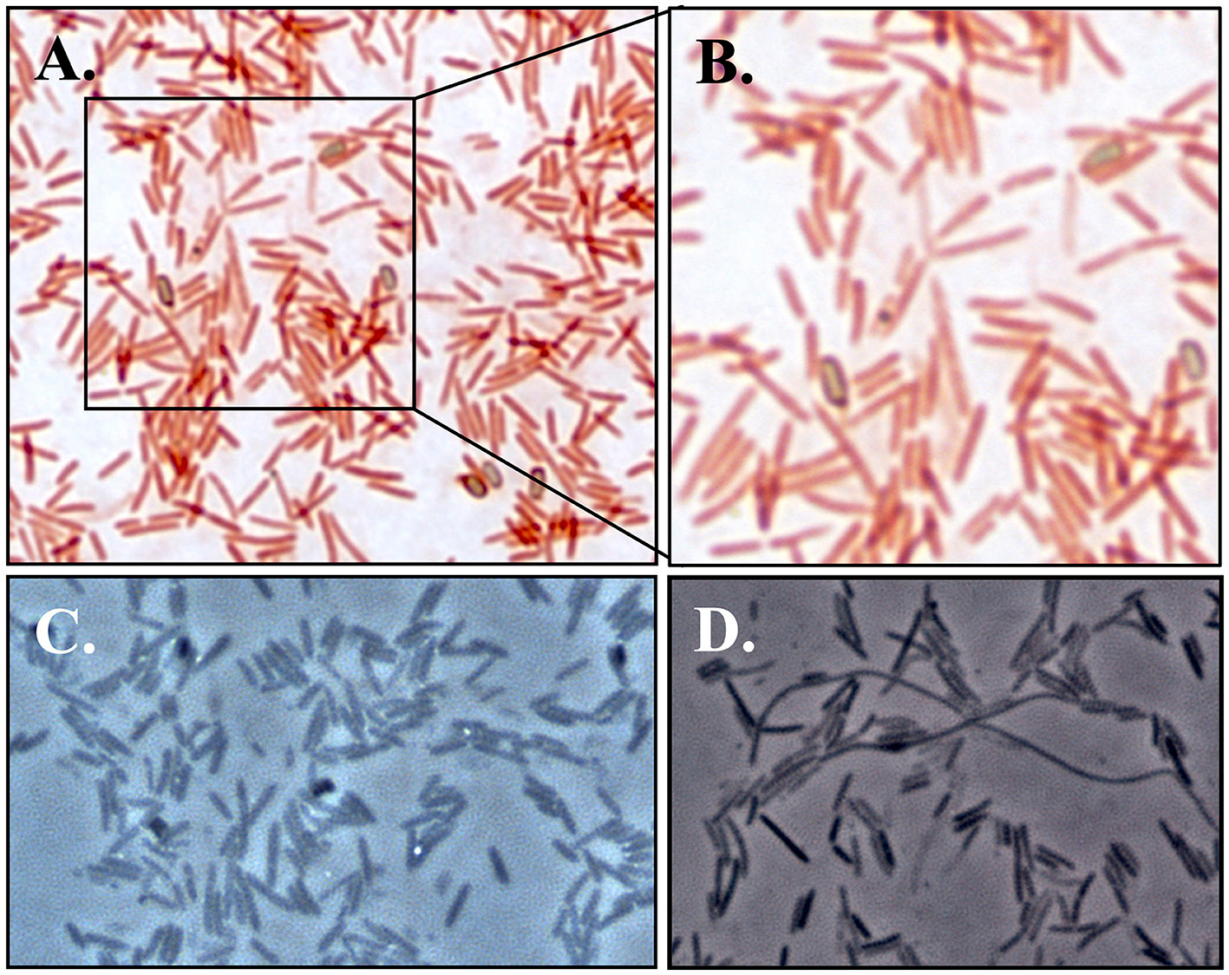
Light microscopy images of strain F6_2S_P_1^T^. **(A)** Bright field images at 1000X magnification with staining with safranin and malachite green for endospore visualization; **(B)** close-up of A featuring multiple endospores which are beginning to transition back to vegetative cells, as well as an endospore within a vegetative cell; **(C)** phase-contrast microscopy (1000X) of strain F6_2S_P_1^T^ showing the presence of endospores; **(D)** phase-contrast microscopy (1000X) of strain F6_2S_P_1^T^ showing the presence of elongated Spaghetti-like cells.

**Figure 5:**
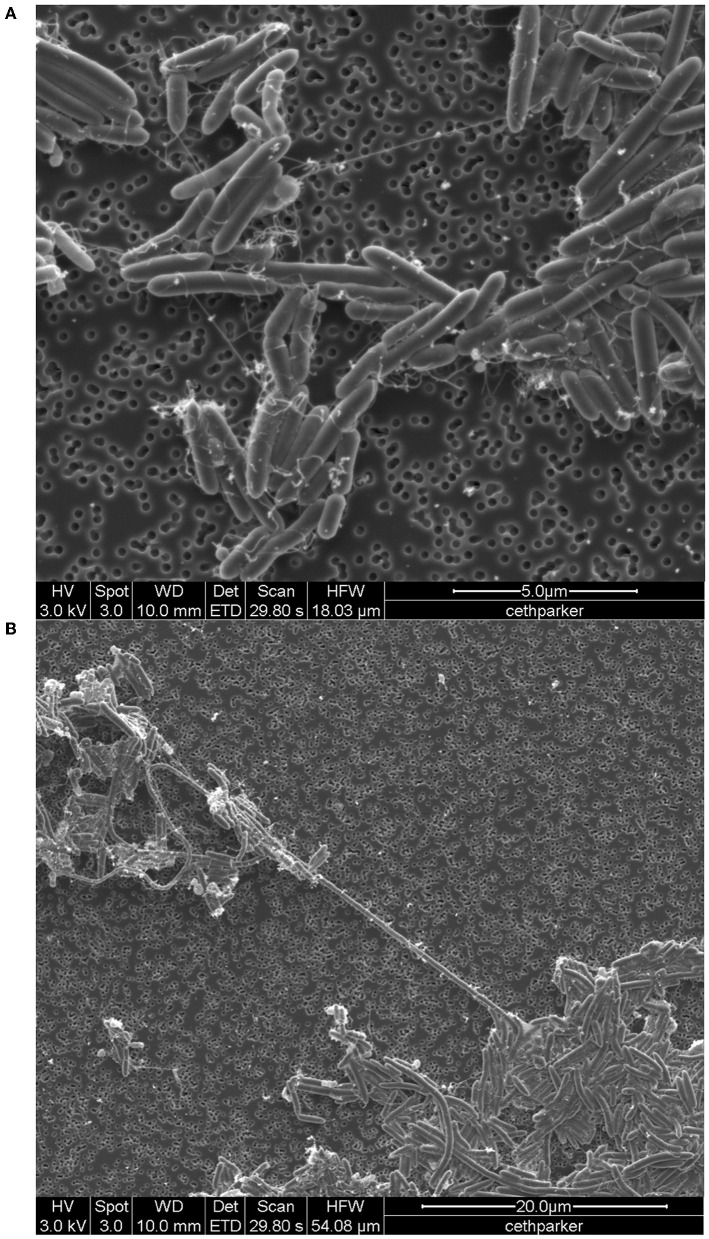
SEM image of *Cohnella* sp. F6_2S_P_1^T^, **(A)** with the visible presence of flagella and **(B)** showing long “spaghetti-like” cells.

Light microscopy and staining with safranin and malachite green show that cells are rod-shaped and vary extensively in length and that strain F6_2S_P_1^T^ forms ellipsoidal/oval endospores which quickly convert back to vegetative cells (Figure 4). SEM imaging reveals the presence of peritrichous flagella attached to F6_2S_P_1^T^ cells (Figure 5A) and confirms the presence of elongated cells (Figure 5B).

Differentiating biochemical characteristics of *Cohnella* sp. F6_2S_P_1^T^ with other *Cohnella* species are depicted in Table 3. *Cohnella* sp. F6_2S_P_1^T^ grew at temperatures between 15 and 37°C, between pHs 6 and 8, and from 0 to 3% NaCl. Colonies were weakly oxidase-positive and catalase-negative. Catalase positive in *C. ginsengisoli, C. plantaginis*, and *C. capsici*; absence of growth at 3% NaCl and production of oxidase in *C. ginsengisoli*; absence of endospore formation in *C. rhizosphaerae*; nitrate reduction not seen in *C. plantaginis* and *C. capsica* were some of the phenotypic characteristics that could differentiate *Cohnella* sp. F6_2S_P_1^T^ from other *Cohnella* species.

**Table 3:** Phenotypic characteristics of strain F6_2S_P_1^T^ and closely related species of genus *Cohnella*.

**Characteristic**	**1**	**2**	**3**	**4**	**5**
Growth temperature (°C)	15–37	15–45	10–40	10–45	4–45
Growth pH	6.0–8.0	5.5–9.5	5.0–9.0	5.0–8.0	5.0–8.0
Growth in the presence of 3% NaCl	+	+	–	+	+
Endospore formation	+	–	+	+	+
Motility	+	–	+	+	+
Catalase	–	–	+	+	+
Nitrate reduction	+	+	+	–	–
Oxidase	w	w	+	–	–
Dextrin	+	N.D.	N.D.	+	+
**Assimilation of:**					
Alanine	+	–	–	+	N.D.
Histidine	+	–	–	+	N.D.
Serine	+	–	–	+	N.D.
Sucrose	+	w	–	+	+
L-Arabinose	+	w	+	–	N.D.
Melibiose	+	w	+	—	N.D.
D-Fructose	+	w	N.D.	+	+
Inositol	+	+	—	N.D.	+
Lactose	+	–	N.D.	+	+
**Acid production from:**					
Maltose	+	–	N.D.	+	–
Sucrose	+	–	N.D.	+	+
D-fructose	+	+	N.D.	+	+
D-ribose	+	+	N.D.	+	+
*N*-acetyl glucosamine	+	+	N.D.	+	+
Lactose	+	–	N.D.	+	+
Trisodium citrate	–	+	N.D.	+	–

Strains: 1, F6_2S_P_1^T^ (*Cohnella hashimotonis sp. nov*.); 2, *Cohnella rhizosphaerae* CSE-5610^T^ (Kampfer et al., 2014); 3, *Cohnella ginsengisoli* DSM 18997^T^ (Kim et al., 2010); 4, *Cohnella plantaginis* YN-83^T^ (Wang et al., 2012); and 5, *Cohnella capsici* YN-59^T^ (Wang et al., 2015). w, weakly positive; +, positive; –, negative; N.D., not determined.

Major fatty acids (more than 5% of the total) are C16:0, iso-C15:0, iso-C16:0, and anteiso-C15:0 (Table 4). Differences in fatty acid profile of strain F6_2S_P_1^T^ with regard to *C. rhizosphaerae* CSE-5610^T^, *Cohnella ginsengisoli* DSM 18997^T^, *C. plantaginis* YN-83^T^, and *C. capsici* YN-59^T^ are shown in Table 4. *C. rhizosphaerae* showed >25% of C16:0 saturated fatty acid, whereas other *Cohnella* species exhibited <10% production. Absence of branched fatty acid such as iso-C14:0 in *Cohnella* sp. F6_2S_P_1^T^ and in *C rhizosphaerae* could be used to differentiate the novel species described in this study. However, production of iso-C16:0 and anteiso-C15:0 was very high in *Cohnella* sp. F6_2S_P_1^T^ when compared to other described *Cohnella* species.

**Table 4:** Percentage of total cellular fatty acids for strain F6_2S_P_1^T^ and closely related species of genus *Cohnella*.

**Fatty acids**	**1**	**2**	**3**	**4**	**5**
**Saturated**					
C_12:0_	–	3.3	–	–	–
C_14:0_	2.0	5.6	3.1	1.6	1.6
C_16:0_	4.9	25.6	6.7	8.9	7.7
**Branched**					
iso-C_14:0_		–	8.2	4.0	4.1
iso-C_15:0_	10.5	10.2	12.2	9.7	11.3
iso-C_16:0_	23.1	12.2	15.0	19.2	18.6
iso-C_17:0_	1.1	–	–	Tr	1.2
anteiso-C_15:0_	51.7	36.5	48.9	45.3	44.3
anteiso-C_17:0_	2.2	6.7	1.8	3.5	3.1
**Unsaturated**					
C_18:1_-ω9c	1.1	–	–	1.5	1.4

Strains: 1, F6_2S_P_1^T^ (*Cohnella hashimotonis sp. nov*.); 2, *Cohnella rhizosphaerae* CSE-5610^T^ (Kampfer et al., 2014); 3, *Cohnella ginsengisoli* DSM 18997^T^ (Kim et al., 2010); 4, *Cohnella plantaginis* YN-83^T^ (Wang et al., 2012); and 5, *Cohnella capsici* YN-59^T^ (Wang et al., 2015). –, Not detected, Tr, trace amount (<1%).

The polar lipid profile was composed of diphosphatidylglycerol, phosphatidylglycerol, phosphatidylethanolamine, three aminophospholipids, a phospholipid, and minor amounts of unidentified polar lipid (Supplementary Figure S2). This profile is in agreement with other described members of the *C. ginsengisoli* clade (Kim et al., 2010; Kampfer et al., 2014; Wang et al., 2015).

The quinone system contained menaquinones MK-7 (98%) and MK-8 (2%). The cell-wall peptidoglycan of strain F6_2S_P_1^T^ contained meso-diaminopimelic acid as the diamino acid, which is the case for other described members of the *C. ginsengisoli* clade where that data are reported. All other described members of the *C. ginsengisoli* clade also contain MK-7 as the dominant menaquinone (Kim et al., 2010; Kampfer et al., 2014; Wang et al., 2015). Descriptions of *C. capsici* and *C*. *plantaginis* do not report low abundance menaquinones, but the quinone system of F6_2S_P_1^T^ is similar to that of *C. rhizosphaerae* (99.8% MK-7, 0.2% MK-8), while *C. ginsengisoli* has 93% MK-7 and 7% MK-6.

### Comparative genomic analysis of *C. hashimotonis* and the *C. ginsengisoli* clade

**Figure 6:**
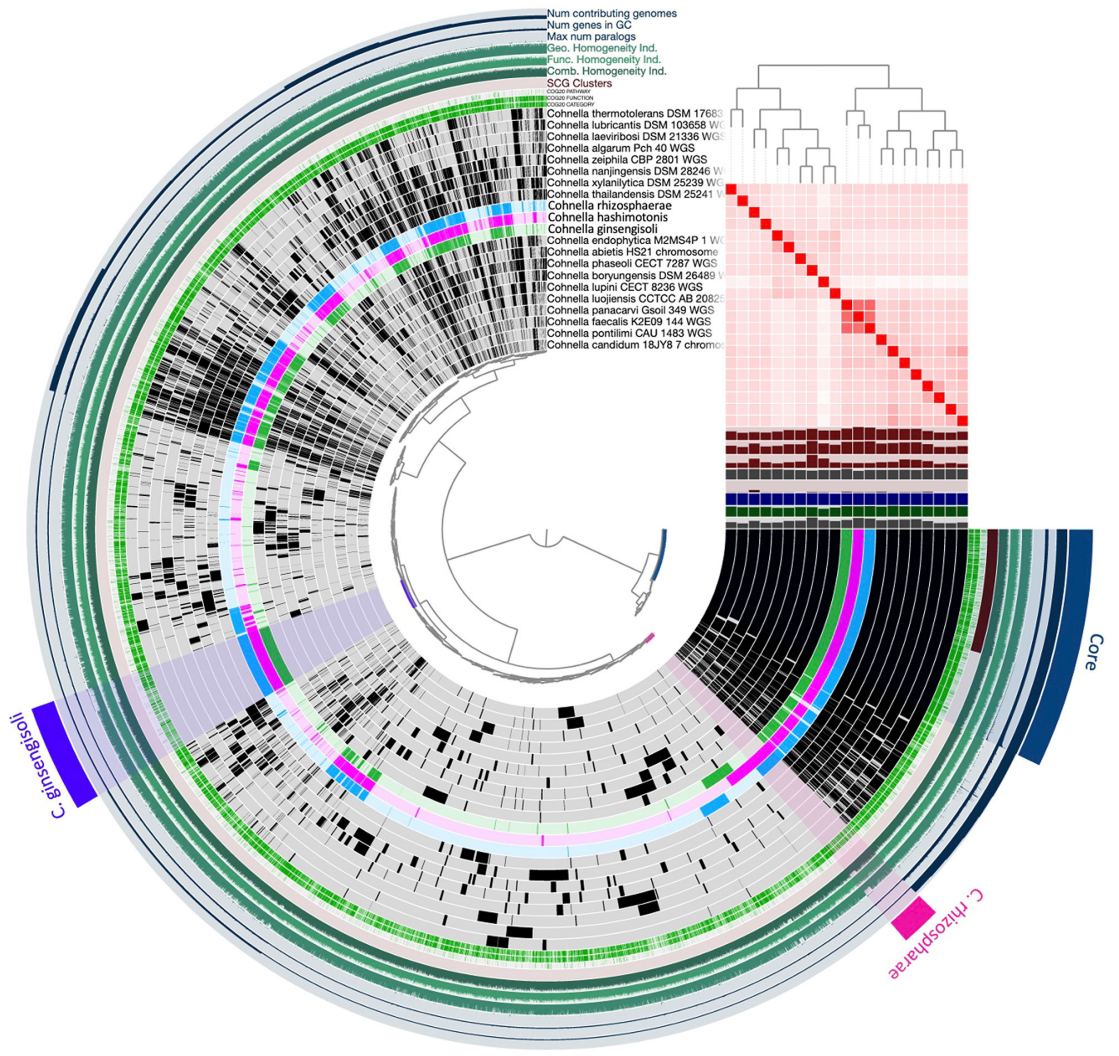
Pangenomic analysis of the genus *Cohnella*, including C. hashimotonis sp. nov. (pink) as well as its previously unsequenced closest relatives C. rhizosphaerae (blue) and C. ginsengisoli (green).

The genome of strain F6_2S_P_1^T^ contained 309 genes not aligned with other species of *Cohnella*, named or undescribed (Figure 6). Of these genes, 121 of them received an annotation from at least one predictive database. Annotated genes specific to strain F6_2S_P_1^T^ were dominated by the COG pathways of carbohydrate and/or amino acid transport and metabolism (27 genes), transcription (10 genes), cell wall/membrane/envelope biogenesis (11 genes), and signal transduction mechanisms (+ transcription) (18 genes). The large number of carbon-cycling metabolic genes found in the genome of strain F6_2S_P_1^T^ within *Cohnella* is unsurprising, given the diverse nature of the carbon substrates which strain F6_2S_P_1^T^, *C. capsici*, and *C. plantaginis* can metabolize compared to the fairly limited substrates utilized by *C. rhizosphaerae* and *C. ginsengisoli* (Table 3).

Because *C. capsici* and *C. plantaginis* are both characterized as fixing N_2_, the annotations for the genome of strain F6_2S_P_1^T^ were searched extensively for genes having to do with nitrogen fixation, as well as blastn and blastx searches for key genes involved in N_2_ fixation. However, despite the presence of the gene nifU in most species of the genus *Cohnella*, there was no indication that strain F6_2S_P_1^T^ encodes for N_2_ fixation.

Other genes of interest predicted by annotation on the RAST platform include multiple genes for endospore formation and for the plant growth hormone auxin, genes which are likely shared by many members of the genus *Cohnella*, which belongs to the order *Bacillales* (including many known spore formers) and which are common plant endophytes. BGCs which might produce secondary metabolites were predicted in the genome of strain F6_2S_P_1^T^ via the antiSMASH pipeline, including two lassopeptides, two non-ribosomal peptide synthetase clusters, one agrD-like cyclic lactone autoinducer peptide, one protein cluster, and one heterocyst glycolipid synthase-like polyketide synthase. All are uncharacterized except the two lassopeptides; these are moderate matches to the lasso peptide paeninodin which was characterized from the species *Paenibacillus dendritiformis* (Zhu et al., 2016). Paeninodin and BGCs showing similarity to it have been predicted in multiple *Bacillus* and *Paenibacillus* endophyte species and are suspected to have biocontrol properties that combat plant pathogens (Muñoz et al., 2022; Semenzato et al., 2022). The genomes of *C. ginsengisoli, C. rhizosphaerae*, and strain F6_2S_P_1^T^ all contained 2–3 predicted BGCs for paeninodin, with *C. ginsengisoli* containing one that is a 100% match, while *C. nanjingensis* (the most closely related species outside the *C. ginsengisoli* clade) contained a single paeninodin match. The only other match in any of these species to a known BGC is a predicted cluster in the *C. rhizosphaerae* which is a moderate match to ectoine. Other differences include multiple cyclic lactone autoinducer peptide BGCs predicted in the genome of *C. nanjingensis* (vs. a single one in all other tested genomes), and a siderophore predicted in the genome of *C. rhizosphaerae*. Strain F6_2S_P_1^T^ was the only genome in the genus *Cohnella* which a heterocyst glycolipid synthase-like polyketide synthase was detected; these BGCs are used by cyanobacteria to compartmentalize N_2_ fixation and are activated in low-nitrogen conditions (Campbell et al., 1997), but as strain F6_2S_P_1^T^ does not contain a full suite of genes for N_2_ fixation and the cluster is not a strong match, it is likely a producer of some kind of potentially uncharacterized lipid compound. No genes for antimicrobial resistance or virulence were predicted by the ABRicate pipeline.

The entire closely related branch of which strain F6_2S_P_1^T^ is a member, which we term the *C. ginsengisoli* clade (due to its basal position and status as the first officially described species within the clade), contained 322 gene clusters not found in any other publicly available *Cohnella* genome. All members of this clade in addition to strain F6_2S_P_1^T^ were originally isolated from plant tissue or the rhizosphere environment, including *C. ginsengisoli* (isolated from soil in which ginseng was grown, Kim et al., 2010), *C. rhizosphaerae* (isolated from healthy maize seed tissue, Kampfer et al., 2014), *C. capsici* (isolated from *Capsicum* rhizosphere, Wang et al., 2015), *C. plantaginis* (isolated from plantain rhizosphere, Wang et al., 2012), *Cohnella* sp. OV330 (isolated from poplar rhizosphere, JGI, 2015), and *Cohnella* sp. GbtcB17 (isolated from *Ginko biloba* root tissue, Zou et al., 2021). It is speculated that strain F6_2S_P_1^T^ might have originated from the plants grown in ISS. Since shotgun metagenomics of plants associated samples (tissue, water, and soil) were not available, the source of this novel strain isolated from the WHC location could not be confirmed. *Cohnella* was one of the top 30 genera recently detected via 16S amplicon sequencing of plant tissue of chili peppers grown in the Advanced Plant Habitat from sterilized seeds aboard the ISS, though it was not a top genus detected in artificial soil medium (Khodadad et al., 2022). *Cohnella* was also not a dominant member of the microbiome of Outredgeous lettuce grown aboard the ISS (Khodadad et al., 2020). However, since in these ISS studies, a 250-bp 16S rRNA gene amplicon was used for the microbial diversity analyses, *Cohnella* species might have not been identified appropriately.

Functional enrichment analysis for the *C. ginsengisoli* clade compared to other *Cohnella* species using the COG20 and KOfam databases revealed hundreds of statistically significantly enriched predicted functions (*p* < 0.05 with FDR adjustment) annotating one or more gene clusters, including 11 functions which were detected in all *C. ginsengisoli* clade members (Supplementary Table S1) but in none of the rest of the genus. Most notably, *C. ginsengisoli* clade species were enriched in genes for heme oxygenase and for a bacteriophytochrome (annotated on NCBI as diguanylate cyclase), which were detected using both COG20 and KOfam database annotation. Consensus protein sequences for genes related to these functions, when aligned to the NCBI database, most closely match to the genome of *Paenibacillus chitinolyticus* (after matches to members of the *C. ginsengisoli* clade); heme oxygenase and diguanylate cyclase are located one after the other in the assembled chromosome of this species (NZ_CP026520.1). Heme oxygenase (HO-1) breaks down heme and releases iron and is a regulator of intracellular iron availability in plants and animals (Singh and Bhatla, 2022). Enhanced HO-1 expression in plants and animals is also associated with stress response as the resulting biliverdin and bilirubin scavenge ROS species (Singh and Bhatla, 2022); the bacterial species *Paenibacillus polymyxa* applied as a probiotic induces increased intestinal HO-1 expression as part of its anti-oxidant effects on gut health in animals (Wu et al., 2019). In bacteria, expression and production of heme oxygenase are associated with iron scavenging within a host, and heme oxygenase expression can have either beneficial anti-oxidant or enhanced pathogenic effects on plant or animal hosts (Richard et al., 2019). In non-photosynthetic bacteria, heme oxygenase also produces biliverdin which is used as the chromatophore in bacteriophytochromes (Takemoto et al., 2019). It is also possible that this HO-1/diguanylate cyclase complex acts as an oxygen sensor (Martínková et al., 2013). Other functions significantly enriched in this clade include stress response genes such as heat shock protein DnaJ. KEGG pathway analysis predicted two functional pathways in all *C. ginsengisoli* clade species which were not present in any other *Cohnella* species: a pathway for carbon fixation (incomplete reductive citrate cycle) and a pathway for plant terpenoid biosynthesis (specifically beta-carotene biosynthesis).

**Figure 7:**
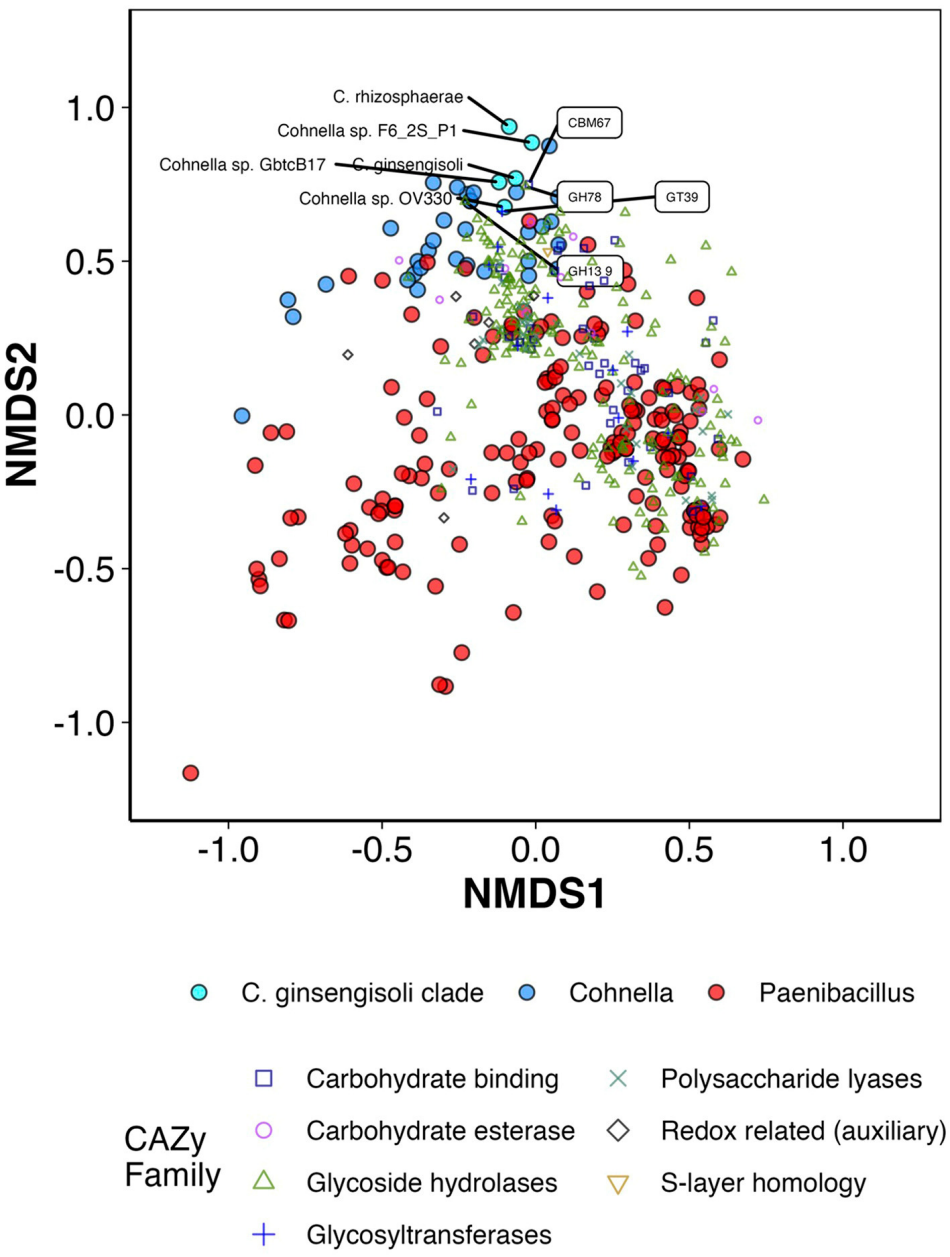
First two axes of NMDS ordination (standardization = “range,” distance = “Bray-Curtis,” maxit = 500, k = 3, stress = 0.129, R^2^ = 0.98) for counts of different predicted CAZymes in the genomes of *Cohnella* and Paenibacillus species. Also present in the figure are CAZyme vector correlations with axes 1 and 2 (not original coordinates) via envfit/Pearson's correlation with fdr correction + p < 0.01. Species belonging to the C. ginsengisoli clade and the top 4 CAZyme families/subfamilies positively correlated with axis 2 are labeled.

Predictive analysis of the presence of CAZymes (carbohydrate-active enzymes, Drula et al., 2022) was followed by ordination (Figure 7; Supplementary Figure S3) and heatmap generation (Supplementary Figure S4). Both heatmaps and ordinations show that strain F6_2S_P1^T^ and its closest relatives cluster tightly together, along with *C. fermenti* (isolated from a fermented vegetable, Lin et al., 2020)*, C. phaseoli* (isolated from the root nodule of a legume species, Garcia-Fraile et al., 2008)*, C. zeiphila* (isolated from corn tissue, Johnson et al., 2021), and several undescribed strains; members of the *C. ginsengisoli* clade, therefore, appear to be both taxonomically similar and have similar profiles of carbohydrate-cycling genes. PERMANOVA analysis confirmed that the centroid of the *C. ginsengisoli* clade was significantly different from that of other *Cohnella* and *Paenibacillus* species (*p* < 0.001). One axis out of three in the NMDS ordination clearly differentiates the *C. ginsengisoli* clade and the other *Cohnella* species mentioned above from the majority of *Cohnella* and *Paenibacillus* species (NMDS2, shown in Figure 7; Supplementary Figure S3); therefore, scores correlating CAZyme ordination vectors to this axis using the envfit function were assessed for significance, plotted, and ranked against the axis in question.

Most strongly associated with this axis and the *C. ginsengisoli* clade are the CAZyme families CBM67 and GH78, which were both identified as part of the enzyme α-L-rhamnosidase which alters flavonoids and other rhamnose-containing glycosides such as pectins in plant cell walls (Yadav et al., 2010). Flavonoids are phenolic compounds produced by plants that often have medicinal or flavorful properties. α-L-rhamnosidase cleaves terminal α-rhamnose residues and in doing so helps concentrate and improve the action of flavonoids; α-L-rhamnosidase from different bacterial species is used for many industrial applications such as improving wine aroma, sweetening fruit juice, and increasing concentration and efficacy of antibiotic and anti-cancer compounds **(**Xie et al., 2022**)**. The *C. ginsengisoli* clade and other *Cohnella* species enriched in α-L-rhamnosidase enzyme modules are all confirmed or likely plant symbionts or feed on plant tissue; however, since the majority of *Cohnella* and *Paenibacillus* species appear to have at least some association with plant tissue or rhizosphere, there may be additional promising applications for these particular species in processing plant medicinal or other plant phenolic compounds.

CAZymes negatively associated with the *C. ginsengisoli* clade (and associated mainly with *Paenibacillus* species) include chitosanases (GH46 and CBM12), endo-β-N-acetylglucosaminidase (GH85), and β-1,4-mannosyl-glycoprotein β-1,4-N-acetylglucosaminyltransferase (GT17). Four CAZyme families associated with the *C. ginsengisoli* clade compared to other *Cohnella* and *Paenibacillus* species had an effect size of 0.5 or greater (Kruskal–Wallis); these were maltooligosaccharide-producing α-amylases (CG13_5), chondroitin AC lyase (PL8_3), and alginate lyases (PL34 and PL14_3).

### Presence of *Cohnella* sp. F6_2S_P_1^T^ in ISS metagenomes

Metagenomic reads from ISS surfaces, crew capsule, and ISS HEPA filter particulates (Venkateswaran et al., 2014; Checinska Sielaff et al., 2019; Urbaniak et al., 2022) were mapped to the genome of strain F6_2S_P_1^T^. Most metagenomes had <0.05% of reads that mapped to strain F6_2S_P_1^T^, with a maximum of 0.22% of reads mapping to strain F6_2S_P_1^T^ for a low-read-number sample collected from the cupola of the ISS during Flight 4 of the Microbial Tracking 2 mission series. But upon examination of regions to which the metagenomic reads mapped to strain F6_2S_P_1^T^, 98+% of reads mapped to regions of 16S or 23S rRNA genes and were only considered significant by bowtie2 because of the conserved nature of the 16S and 23S rRNA genes. We therefore concluded that strain F6_2S_P_1^T^ is not dominant in the ISS environmental surfaces or air particulates collected during the Microbial Tracking 1 and 2 mission series. However, molecular microbial diversity-based examination of plant and its associated materials using culturing and metagenomics would allow NASA to trace the source of this strain, since *Cohnella* species were reported to produce hormones that might promote growth of plants. For example, as mentioned above, *Cohnella* was an abundant genus in the microbiome of healthy plant tissue of chili plants grown aboard the ISS (Khodadad et al., 2022).

## Conclusion

We used multiple means of characterization, including phenotypic, chemotaxonomic, phylogenetic, and functional genome-prediction methods to characterize *Cohnella* sp. F6_2S_P_1^T^ and place it within the established taxonomy of the genus *Cohnella*. Based on these data, we propose that this novel species be named *C. hashimotonis*. We also characterized the branch of *Cohnella* within which *C. hashimotonis* is placed and predicted functional characteristics of *C. hashimotonis* and the *C. ginsengisoli* clade as a whole. All species of *Cohnella* closely related to *C. hashimotonis* are from the plant/rhizosphere environment and likely have mutualistic associations with plant roots or other tissue. It is likely, therefore, that *C. hashimotonis* was introduced to the ISS not as a human commensal but as a plant commensal during one of the many plant growth experiments conducted on board. Nevertheless, *C. hashimotonis* can clearly survive in the extreme conditions of the ISS built environment, including radiation exposure, desiccation, low nutrient availability, and microgravity, and is closely related to a number of rhizosphere bacteria; it is therefore a promising candidate for testing as a member of plant-growth-promoting consortia for future plant experiments in outer space.

## Description of *Cohnella hashimotonis* sp. nov.

*Cohnella hashimotonis* (ha.shi.mo.to.nis. masc. n. *hashimotonis* named in honor of Professor Hideo Hashimoto, a food microbiologist from Hiroshima Univ., Japan). Cells are endospore-forming, aerobic, and gram-positive rods, 0.5–0.6 μm in width and 0.8–100+ μm in length. In addition to normal cells, elongated, spaghetti-like cells (Figures 4D, 5B) are observed in light microscopy and SEM images. Colonies are translucent cream-colored, acquiring a pinkish tinge after multiple days of growth, and grow well on R2A but poorly on TSA. Growth occurs at between 15 and 37°C, between pHs 6 and 8, and from 0 to 3% NaCl. Colonies are weakly oxidase-positive and catalase-negative. This species reduces nitrate to nitrite, produces dextrin from starch, and utilizes the following carbon substrates: alanine, histidine, serine, sucrose, L-arabinose, melibiose, D-fructose, inositol, and lactose. The following substances are utilized for acid production: maltose, sucrose, D-fructose, D-ribose, N-acetyl, glucosamine, lactose, and trisodium citrate. Major fatty acids composing *C. hashimotonis* are anteiso-C_15:0_ (51.7%), iso-C_16:0_ (23.1%), and iso-C_15:0_ (10.5%). The polar lipids are diphosphatidylglycerol, phosphatidylglycerol, phosphatidylethanolamine, three aminophospholipids, and a phospholipid. The major quinone system is menaquinones MK-7. The cell-wall peptidoglycan of strain contained meso-diaminopimelic acid as the diamino acid.

*C. hashimotonis* is closely related to *C. rhizosphaerae* and *C. ginsengisoli* based on 16 rRNA gene sequence similarity (<99%). However, the phylogenetic position of the strain F6_2S_P_1^T^ was resolved as a novel species due to gyrB sequence similarity (<94%), ANI (<89%), and dDDH (<35%) analyses with any other sequenced *Cohnella* species. GC content is 58.37%. The type strain F6_2S_P_1^T^ was isolated from the wall behind the Waste Hygiene Compartment, i.e., the space toilet, aboard the ISS, in 2018; its genome is available on NCBI, accession number JAGRPV000000000. The type strain F6_2S_P_1^T^ is submitted to two culture collections (=NRRL B-65657^T^ and DSMZ 115098^T^).

## Data availability statement

The datasets presented in this study can be found in online repositories. The names of the repository/repositories and accession number(s) can be found in the article/Supplementary material.

## Author contributions

NS and KV managed the ISS strain collection and the ISS strain genome sequencing project. AS, VE, and KV conceived and designed the study. VE and AS performed the microbiological experiments and carried out the phenotypic assays. VE performed BioLog based biochemical characterization and chemotaxonomy. FK managed to coordinate in sample collection from ISS and developed sample collection protocols. CM and ND generated shotgun metagenome sequences using ONT platform. NS performed the original taxonomic analysis and *de novo* assembly. AS performed genomic, pangenomic, and metagenomic characterization, long-read assemblies, and light microscopy assays and wrote the manuscript. KV edited the manuscript. CP performed SEM on F6_2S_P_1^T^. All authors read and approved the final manuscript.
