# Supplementary material for: Comparative genomic analysis of Cohnella hashimotonis sp. nov. isolated from the International Space Station
Source: Front Microbiol. 2023 Jun 15;14:1166013. doi: 10.3389/fmicb.2023.1166013 (PMC10308117; doi:10.3389/fmicb.2023.1166013)
Supplement: Supplementary file 1 [file Data_Sheet_1.docx]

**Supplemental tables and Figures:**

Table S1: COG, Kofam, and KEGG functions enriched in the *C. ginsengisoli* clade species but not in other *Cohnella* species

| **gene_clusters_ids** | **COG20_FUNCTION** | **COG20_Accession** | **KOfam** | **Kofam Accession** | **KEGG_Class** | **KEGG Accession** |
| --- | --- | --- | --- | --- | --- | --- |
| GC_00006096 | – | – | – | – | Pathway modules; Energy metabolism; Carbon fixation:  Incomplete reductive citrate cycle, acetyl-CoA => oxoglutarate | M00620 |
| GC_00006898 | Bacteriophytochrome (light-regulated signal transduction histidine kinase) (PDB:2VEA) | COG4251 | two-component system, chemotaxis family, sensor kinase Cph1 [EC:2.7.13.3] | K11354 | – | – |
| GC_00006913 | – | – | two-component system, NtrC family, nitrogen regulation sensor histidine kinase NtrY [EC:2.7.13.3] | K13598 | – | – |
| GC_00007237 | Beta- N-acetylglucosaminidase (LytD) (PDB:6FXO) | COG4193 | – | – | – | – |
| GC_00007257 | – | – | ATP-dependent Clp protease ATP-binding subunit ClpB | K03695 | – | – |
| GC_00007263 | AraC-type DNA-binding domain and AraC-containing proteins (AraC) (PDB:1BL0)!!!Sugar diacid utilization regulator CdaR (CdaR) | COG2207!!!COG3835 | – | – | – | – |
| GC_00007360 | – | – | translocator protein | K05770 | – | – |
| GC_00007401 | – | – | curved DNA-binding protein | K05516 | – | – |
| GC_00007425 | L,D-peptidoglycan transpeptidase YkuD, ErfK/YbiS/YcfS/YnhG family (PUBMED:16647082) | COG3786 | – | – | – | – |
| GC_00007427 | – | – | colanic acid/amylovoran biosynthesis glycosyltransferase [EC:2.4.-.-] | K16703 | – | – |
| GC_00007447 | Signal transduction histidine kinase (BaeS) (PDB:1JOY)!!!PAS domain (PAS) (PDB:2MWG)!!!MHYT domain, NO-binding membrane sensor (MHYT)!!!Two-component response regulator, PleD family, consists of two REC domains and a diguanylate cyclase (GGDEF) domain (PleD) (PDB:1W25) | COG0642!!!COG2202!!!COG3300!!!COG3706 |  |  | – | – |
| GC_00007453 | Heme oxygenase (HemO) (PDB:1J77) | COG3230 | heme oxygenase (biliverdin-IX-beta and delta-forming) [EC:1.14.99.58] | K07215 | – | – |
| GC_00008216 | – | – | – | – | Pathway modules; Biosynthesis of terpenoids and polyketides; Plant terpenoid biosynthesis | M00097 |


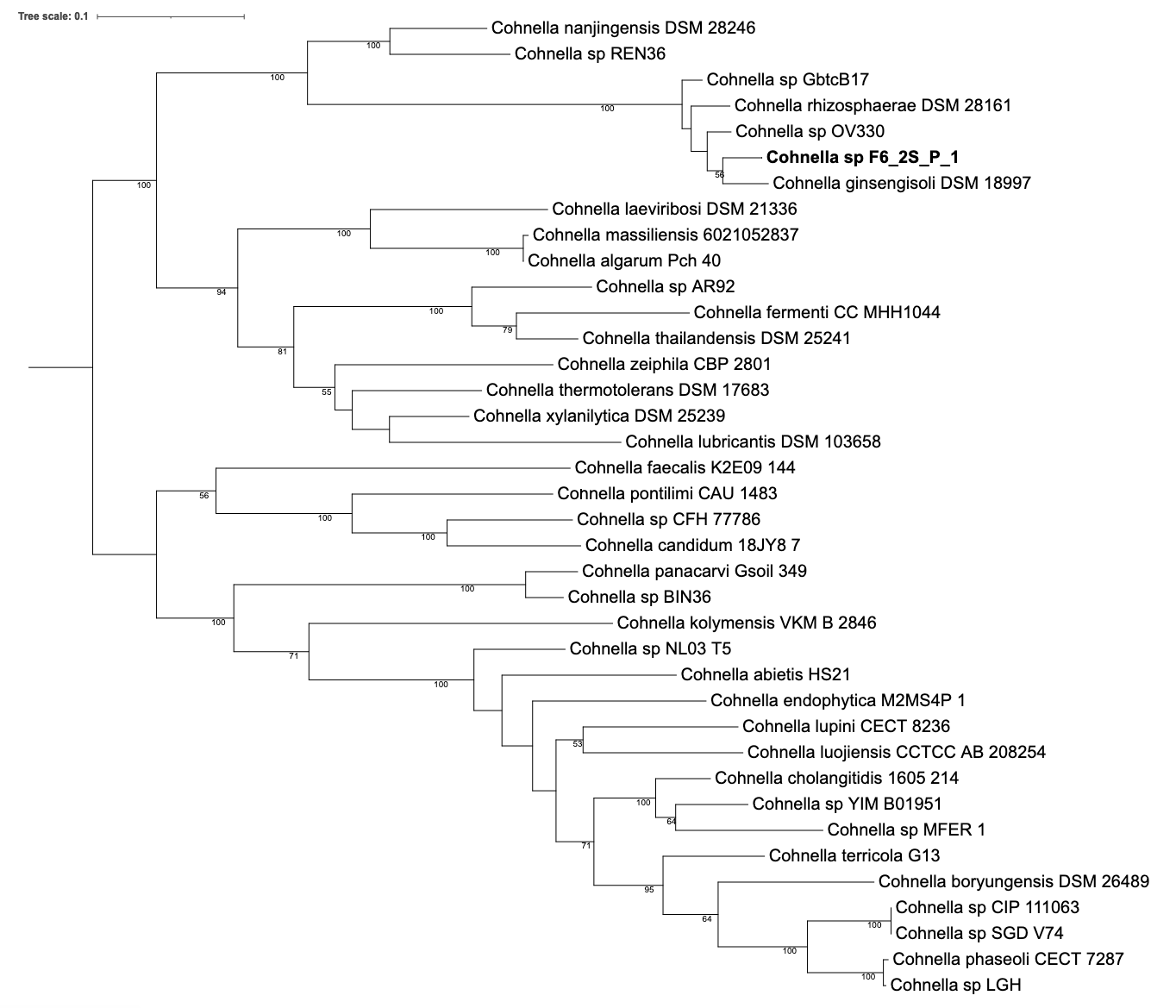


Figure S1: Whole-genome tree of single copy genes extracted from pangenomic analysis, from all publicly available *Cohnella* genomes including unnamed species.


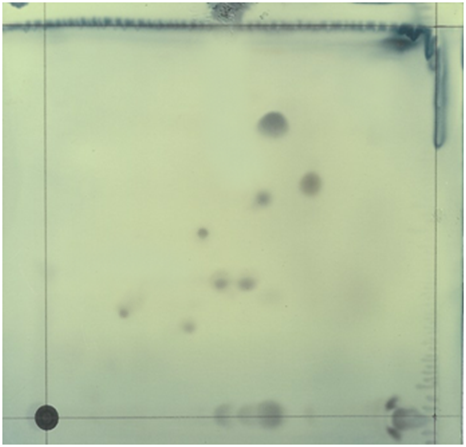


2 ^nd^ Dimension

1^st^ Dimension

*Cohnella* sp. F6_2S_P_1^T^

DPG

PE

PG

L

PL

APL3

APL2

APL1

Figure S2. Polar lipid profile of *Cohnella* sp. F6_2S_P_1^T^

**
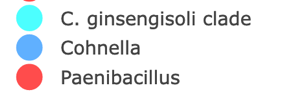

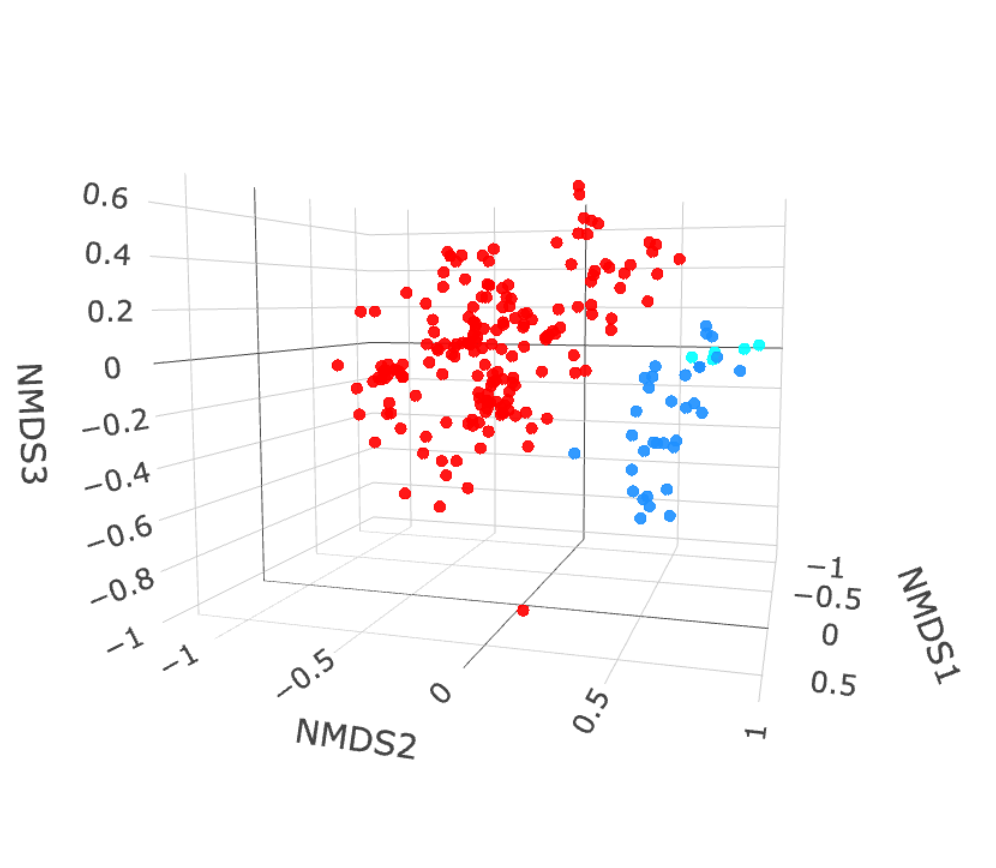
**

Figure S3: NMDS ordination shown in Figure 7 with all 3 dimensions. Axis 3 does not differentiate the *C*. *ginsengisoli* clade, which is at the midpoint for this axis.

**
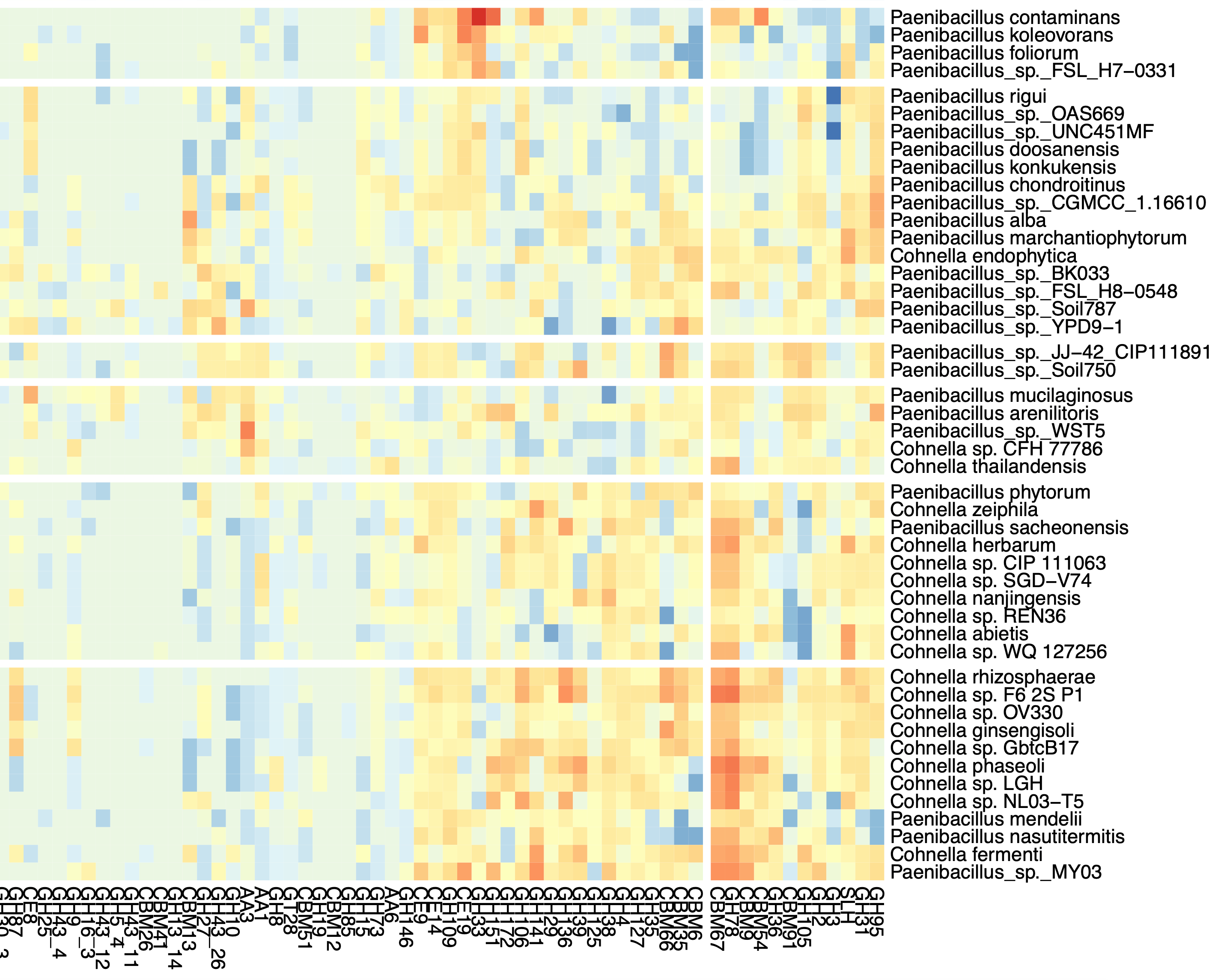
**

Figure S4: Heatmap of frequency of predicted CAZymes in genomes of *Cohnella* and *Paenibacillus* species after robust centroid log-ratio correction.
